# 

**Published:** 1897-01

**Authors:** 


### ILLUSTRATIONS.

PAGE

Acromegaly in the Dog . . . . . . . . . . 446
Cystoma Dermalis . . . . . . . . . . . 227
Hart, John R. . . . . . . . . . . . . 235
Necrosis Totalis of the Scapula . . . . . . . . . 96
Study of Cathartics . . . . . . . . . . . 180
Typhoid Serum Diagnosis . . . . . . . . . . 545

### PERSONAL.

Among those specially interested in the Normandy cattle shown at the National Live-stock Show at Madison Square Garden, New York, in November, we noted on one of the pens a card of our esteemed colleague, Prof. A. Liautard.

Dr. A. S. Wheeler, of New Orleans, has been resting for a period at Asheville, N. C.

Another New York Canine Infirmary was opened on January 1st, under the direction of Veterinarians Huidekoper and Gill.

The December number of the *Horseshoers' Journal* contains articles by Veterinarians Harger, of Pennsylvania, and James Robertson, of Illinois.

Mr. R. A. Pearson, assistant chief of the dairy division of the Department of Agriculture, was an interested visitor at the National Live-stock Show.

Dr. Bryant, of Kansas, arrested in New York City in November for violation of the State veterinary law, pleaded guilty at the hearing and was paroled for sentence.

Since the removal of Dr. E. H. Flood from the post of inspectorship of cattle for foreign shipment, for disobeying Federal instructions in overloading vessels, the duties have been performed by inspector of export meats, Dr. Charles Schauffler. Several applicants for the place are already in the field.

Drs. Adams, Pearson, Hoskins, and McAnulty spoke encouraging words to the class assembled in December at the opening of the Philadelphia School of Anatomy and Scientific Farriery at the Veterinary Department of the University of Pennsylvania. Dr. Hoskins will address the students' society in January.

Dr. Davison, of Millbrook, N. Y., exhibited his Shropshire ram Ringleader in the show circuit from Syracuse to St. Louis and back to the Madison Square Garden Show without the loss of a first prize in any of the shows.

Veterinarian A. S. Alexander, of Evanston, Ill., was a special contributor to the Christmas number of the *Breeders' Gazette.*

Dr. M. E. Knowles has returned to Butte City, Mont., where he will engage in private practice.

Veterinarian W. S. Manley, of Arkansas City, reports the removal of a misplaced molar from the base of the right ear in a two-year-old filly.

Dr. C. C. Cattanach, while abroad during the past summer, had with him one of his famous mules, Carmencita, with which he travelled over a goodly portion of Great Britain.

Veterinarian R. W. Carter accompanied the running horses of Mr. Pierre Lorillard on their campaigning tour through England.

An interesting group noted at the recent Madison Square Garden Show was Secretary Dr. G. Howard Davison, of the National Live-stock Association; Veterinary Inspector R. S. Huidekoper; Prof. H. D. Gill, of the New York Veterinary College, and Editor W. Horace Hoskins, of the JOURNAL.

Dr. Dowd, of Kansas City, has been transferred by the Bureau of Animal Industry to an inspectorship of the Gray's Ferry abattoir in Philadelphia.

The Philadelphia School of Anatomy and Scientific Horseshoing opened its third session on December 1st. The instruction will be given at the Veterinary Department of the University of Pennsylvania, and the valuable services as lecturers have again been obtained of Drs. Harger and Adams.

Dr. F. H. Miller has located on Forty-second Street, New York City, where he will give sole attention to canine practice.

Dr. R. S. Huidekoper was an exhibitor in the Brooklyn Kennel Show of his famous bull terriers, and fresh laurels were added to their former victories.

Among those interested in porcine breeding we note Messrs. Butler, of Mississippi, and Gill, of New Jersey.

Quite a group of veterinarians watched the proceedings in court of the test of the law in the case of Dr. Bryant, arrested in New York City. The decision sustaining the law is to be followed by proceedings against a large number of non-registered violators in New York County and other sections of the Empire State. Laws that are openly violated are worse than no laws at all, for they breed contempt for statutory powers, and we trust that prompt punishment will be meted out to these parasites of the profession.

Dr. R. O. Lusson, of Ardmore, Pa., has received an appointment as an inspector under the Bureau of Animal Industry, and has been assigned to Buffalo.

Dr. Tait Butler has severed his connection with the Agricultural College of Mississippi, and Dr. Joseph C. Robert, graduate of the University of Pennsylvania, has succeeded him as Professor of Veterinary Science in the school.

State Veterinarian Leonard Pearson has become an active member of the State Grange in the Keystone State.

Veterinarians Williams, of Ithaca, N. Y., and Noack, of Reading, Pa., were on the sick list in December, but are now in active pursuit of their labors.

President Ridge, of the Pennsylvania State Veterinary Medical Association, will present a paper at the February meeting of the Keystone Veterinary Medical Association.

Dr. James T. McAnulty, Secretary of the Pennsylvania Association of Master Shoers, which is now a chartered organization, secured favorable indorsement by the Keystone Veterinary Medical Association of the bill to require registration of master and journeymen shoers, which will be presented at the next session of the State Legislature.

Dr. William Tag, of Philadelphia, continues on the sick list, and is at times unable to pursue his daily professional duties.

The invention of the new military bridle and bit by Veterinarian Austin Peters, lieutenant of the State Militia of Massachusetts, is attracting the attention of all the leading horse-journals of the land.

Bulletin 62 of Purdue Experiment Station, entitled "The Udder of the Cow," has just been issued by Prof. C. S. Plumb.

Dr. James K. Thompson, of Pueblo, Col., has left for a trip to Ireland.

At a meeting of the New Jersey State Sanitary Association, held at Lakewood on December 11th, papers were presented by Dr. Wm. Herbert Lowe, of Paterson, on "Infectious Diseases Among Animals;" by Edward B. Voorhees, Ph.D., of New Brunswick, on "Milk: How it should be Collected, Transported, and Stored;" and by George W. McGuire, State Dairy Commissioner, on "Inspection of Dairies." Dr. Wm. H. Lowe was elected a member of the New Jersey State Sanitary Association at this meeting.

Messrs. Carter and Waugh, of Pittsburg, will add a light ambulance to their hospital and will manage it on the patrol system, with horses in readiness to move at a moment's notice.

Dr. Hugh F. Doris, a graduate of the American Veterinary College, later a student of human medicine for three years, is now taking a course of law at the University of West Virginia. He has succesfully filled at the last college the *rôle* of captain and trainer of the foot-ball team.

Dr. Joseph M. Good, of Chattanooga, Tenn., is planning for a milk- and meat-inspectorship for his city.

Dr. Leonard Pearson addressed the Students' Society at the Veterinary Department of the University of Pennsylvania on December 18, 1896.

Dr. A. F. Peters, of Lincoln, addressed the December meeting of the Nebraska Jersey Cattle-breeders' Association on "How to Replenish the Dairy-herd." Dr. J. S. Anderson, of Seward, reported his experience with an outbreak of "Texas Fever" in a herd shipped from Arkansas to Nebraska.

Dr. J. C. McNeil, city veterinarian of Pittsburg, was a recent visitor to Philadelphia, where, with other officials of the Smoky City, an inspection of the police and fire departments was made.

Dr. Robert Formad, of Philadelphia, recently completed a short brochure on the more prominent animal diseases transmissible to man.

Robert W. Ardary, V.S., is constructing a veterinary infirmary in Pittsburg with all modern conveniences.

Veterinarian H. J. Hancock, of Pittsburg, is spending the winter at Canfield, Ohio.

Pennsylvania possesses five brothers in the active practice of veterinary science, who are successively the ages of 77, 72, 65, 63, and 61. They are well-known, successful practitioners, and honored by the communities where they reside, respected by all who know them, and much loved for their honorable methods as members of the veterinary profession.

The Governor of Massachusetts has appointed Dr. Austin Peters a member of the Cattle Commission in place of Dr. F. H. Osgood, resigned. Mr. Leander Herrick has resigned the post of Secretaryship of the Commission, and the official reorganization of this body has resulted in the selection of Dr. Austin Peters, Chairman, and Dr. John M. Parker, Secretary.

Veterinarian Waugh, of Pittsburg, was recently detailed by State Veterinarian Pearson to examine a suspected tuberculous herd of cattle in Westmoreland County.

